# Characterization and classification of lupus patients based on plasma thermograms

**DOI:** 10.1371/journal.pone.0186398

**Published:** 2017-11-17

**Authors:** Nichola C. Garbett, Guy N. Brock, Jonathan B. Chaires, Chongkham S. Mekmaysy, Lynn DeLeeuw, Kathy L. Sivils, John B. Harley, Brad H. Rovin, K. B. Kulasekera, Wael N. Jarjour

**Affiliations:** 1 James Graham Brown Cancer Center, Department of Medicine, University of Louisville, Louisville, KY, United States of America; 2 Department of Bioinformatics and Biostatistics, School of Public Health and Information Sciences, University of Louisville, Louisville, KY, United States of America; 3 Arthritis and Clinical Immunology Program, Oklahoma Medical Research Foundation, Oklahoma City, OK, United States of America; 4 U.S. Department of Veterans Affairs Medical Center, Cincinnati, OH, United States of America; 5 The Center for Autoimmune Genomics and Etiology, Department of Pediatrics, Cincinnati Children’s Hospital Medical Center & University of Cincinnati, Cincinnati, OH, United States of America; 6 Nephrology Division, The Ohio State University Wexner Medical Center, Columbus, OH, United States of America; 7 Division of Rheumatology and Immunology, Department of Internal Medicine, The Ohio State University Wexner Medical Center, Columbus, OH, United States of America; Peking University First Hospital, CHINA

## Abstract

**Objective:**

Plasma thermograms (thermal stability profiles of blood plasma) are being utilized as a new diagnostic approach for clinical assessment. In this study, we investigated the ability of plasma thermograms to classify systemic lupus erythematosus (SLE) patients versus non SLE controls using a sample of 300 SLE and 300 control subjects from the Lupus Family Registry and Repository. Additionally, we evaluated the heterogeneity of thermograms along age, sex, ethnicity, concurrent health conditions and SLE diagnostic criteria.

**Methods:**

Thermograms were visualized graphically for important differences between covariates and summarized using various measures. A modified linear discriminant analysis was used to segregate SLE versus control subjects on the basis of the thermograms. Classification accuracy was measured based on multiple training/test splits of the data and compared to classification based on SLE serological markers.

**Results:**

Median sensitivity, specificity, and overall accuracy based on classification using plasma thermograms was 86%, 83%, and 84% compared to 78%, 95%, and 86% based on a combination of five antibody tests. Combining thermogram and serology information together improved sensitivity from 78% to 86% and overall accuracy from 86% to 89% relative to serology alone. Predictive accuracy of thermograms for distinguishing SLE and osteoarthritis / rheumatoid arthritis patients was comparable. Both gender and anemia significantly interacted with disease status for plasma thermograms (p<0.001), with greater separation between SLE and control thermograms for females relative to males and for patients with anemia relative to patients without anemia.

**Conclusion:**

Plasma thermograms constitute an additional biomarker which may help improve diagnosis of SLE patients, particularly when coupled with standard diagnostic testing. Differences in thermograms according to patient sex, ethnicity, clinical and environmental factors are important considerations for application of thermograms in a clinical setting.

## Introduction

According to the Autoimmune Diseases Coordinating Committee, as many as 24 million people in the US are afflicted with autoimmune disease [1]. Diagnosis of autoimmune disease is particularly difficult because of highly diverse clinical manifestations. Systemic lupus erythematosus (SLE), a prototypic autoimmune disease that is heterogeneous in its presentation, is diagnosed based on clinical history, physical exam and laboratory studies including serological markers. Serological markers are problematic since the ones that are most sensitive are the least specific. This makes early diagnosis difficult and can result in a delay in important treatment. There has been a significant push to develop biomarkers that can accurately establish a diagnosis of SLE, evaluate disease activity, predict prognosis and guide therapy. Despite some promising studies deserving of further attention, few have been validated to-date [2–5]. New diagnostic approaches are therefore of critical importance for both diagnosis and monitoring of SLE and SLE-related disease.

One potential source of multi-purpose diagnostic biomarkers is differential scanning calorimetry (DSC) profiles (or thermograms) [6–21]. Thermograms indicate the heat change (excess specific heat capacity) in a fluid sample as it is heated, corresponding to the structural changes in the molecular constituents of the fluid as a function of temperature (e.g., protein denaturation). DSC thermograms have been successfully used as a diagnostic tool for the characterization of human diseases, including cervical cancer [11], breast cancer [14], colorectal cancer [13], multiple myeloma [15], brain tumors [8, 9], chronic obstructive pulmonary disease [12] and early renal function decline in type 1 diabetes patients [10]. Most relevantly, Garbett et al. [6] previously illustrated differences between average thermograms in a small sample of healthy controls, SLE patients, rheumatoid arthritis (RA) patients, and Lyme disease patients. Fish et al. [22] extended these findings to a sample of 300 SLE patients and 300 non SLE controls, demonstrating that thermograms could classify SLE patients versus non SLE controls with similar accuracy to that based on immunological based markers. However, none of the aforementioned studies developed approaches for applying thermograms to *enhance* current diagnostic approaches for a given disease. Further, few of the studies have reported on the potential heterogeneity of thermograms along important demographic, clinical and environmental factors.

In this study, we examine thermograms as a diagnostic tool in SLE by applying a recently developed classification algorithm to thermograms based on plasma samples from 300 SLE patients and 300 non SLE controls from the Lupus Family Registry and Repository (LFRR) [23]. Further, we perform a comprehensive exploratory investigation of the heterogeneity among thermograms from SLE patients and non SLE controls, including stratification by important demographic variables, laboratory measurements, and environmental exposures. This exploratory investigation has important implications for the clinical utility of DSC. Lastly, we demonstrate that thermograms combined with SLE immunological markers can improve upon classification based on the serological markers alone.

## Materials and methods

### Patient population

De-identified plasma samples and patient data were obtained from the Lupus Family Registry and Repository (LFRR). The LFRR was established to assemble a large collection of materials and data from lupus patients and controls to enable progress in SLE genetics research. Approved users are able to request access to samples and data from the LFRR to pursue research related to SLE. The establishment, subject composition and operation of the LFRR, including human subjects protections, was recently described [23]. Plasma samples and patient data for 300 patients meeting the revised criteria of the American College of Rheumatology for SLE [24] and 300 non SLE controls matched demographically by sex, ethnicity and age were obtained from the LFRR. A patient is classified as having SLE if four of eleven ACR SLE criteria are present [24] (**S1 Table**). Plasma samples were received in frozen form on dry ice and were kept at -80°C until thawed for DSC analysis. The LFRR data allow for the evaluation of any significant association of differences in thermograms with the ACR SLE criteria, as well as relevant demographic, serologic, and clinical data to evaluate influence of these covariates. Demographic and comorbidity data were obtained by questionnaires as indicated in the LFRR database manual. Use of the LFRR samples and clinical data was reviewed and approved by the University of Louisville Institutional Review Board (IRB# 177.07, 12.0543) in compliance with the Helsinki Agreement.

### DSC sample preparation

Samples were prepared according to our previously published procedure [11]. Briefly, plasma samples (100 μL) were dialyzed against a standard phosphate buffer (1.7 mM KH_2_PO_4_, 8.3 mM K_2_HPO_4_, 150 mM NaCl, 15 mM sodium citrate, pH 7.5) for 24 hours at 4°C in order to achieve normalization of buffer conditions for all samples. Samples were recovered from dialysis and filtered to remove particulates. The final dialysis buffer was also filtered and used for all sample dilutions and as a reference solution for DSC studies.

### Collection of DSC thermograms

DSC data were collected according to our previously published procedure [11]. In designing our experimental approach for the analysis of blood plasma samples we have carefully examined each aspect of the process: blood sample collection and handling; sample preparation for DSC analysis; instrument settings and analysis replicates; data analysis and interpretation. These studies have recently been published [25]. Importantly, we demonstrated that plasma thermograms were robust to all analytical and pre-analytical variables examined. These studies enabled us to adopt a standard protocol for the analysis of clinical samples. Our standard protocol based on the limited availability of sample aliquots and to provide reasonable analysis throughput involved the collection of duplicate scans for each sample and batching of samples to ensure that DSC analysis is completed within a seven day window after initial thawing of each sample batch. For each sample set we examined buffer scans collected at the beginning and end of a sample set and after single or consecutive samples scans and determined acceptable reproducibility and effective cleaning of the instrument chambers. We also compared sample scans collected after a buffer or sample scan and found it is possible to collect consecutive sample scans after extensive rinsing of the instrument chambers. Data were collected using an automated MicroCal VP-Capillary DSC instrument (MicroCal, LLC, Northampton, MA, now a division of Malvern Instruments Inc.). Electrical calibration of the differential power signal and temperature calibration using hydrocarbon temperature standards were performed as part of the manufacturer annual instrument maintenance. Interim instrument performance was assessed using biological standards lysozyme and RNaseA. Dialyzed plasma samples were diluted 25-fold to obtain a suitable protein concentration for DSC analysis. Samples and dialysate were loaded into the instrument autosampler and thermostated at 5°C until analysis. Thermograms were recorded from 20°C to 110°C at a scan rate of 1°C/min with a pre-scan thermostat of 15 minutes, mid feedback mode and a filtering period of 2 seconds. Duplicate thermograms were obtained for each plasma sample and examined to ensure measurements were reproducible and unaffected by data collection on different days. DSC data were analyzed using Origin 7 (OriginLab Corporation, Northampton, MA). Raw data were corrected for the instrumental baseline by subtraction of a suitable buffer scan. Thermograms were normalized for total protein concentration which was determined colorimetrically using the bicinchoninic acid protein assay kit and microplate procedure from Pierce (Rockford, IL), with absorbance readings taken with a Tecan Safire^2^ plate reader (Tecan U.S., Research Triangle Park, NC). Following normalization, thermograms were and corrected for non-zero baselines by application of a linear baseline fit. Final thermograms were the average of duplicate measurements and plotted as excess specific heat capacity (cal/°C.g) versus temperature (°C). Two case samples and six control samples were flagged as poor quality data and removed prior to analysis.

### Statistical analysis of thermograms

Thermograms were first visualized for differences between SLE patients and controls by plotting the mean ± the 5^th^ and 95^th^ percentiles for each group at each temperature. To facilitate interpretation of the thermograms, several summary statistics including shape and feature metrics of the thermograms were calculated [11]. These included principal components (PCs) of the thermograms, total area under the thermogram (range 45–90°C), thermogram peak width at half height, maximum peak height, temperature of the peak maximum (T_max_), maximum excess specific heat capacity ($C_{p}^{ex}$) of the first peak (Peak 1 max $C_{p}^{ex}$), maximum $C_{p}^{ex}$ of the second peak (Peak 2 max $C_{p}^{ex}$), the ratio of (Peak 1 max $C_{p}^{ex}$) / (Peak 2 max $C_{p}^{ex}$), and the first moment temperature T_FM_. The T_FM_ was calculated as follows

$$T_{FM}=\frac{\int_{45}^{90}(TC_{p}^{ex})dT}{\int_{45}^{90}C_{p}^{ex}dT}.$$

Intuitively, the T_FM_ corresponds to a central mass point when considering the thermogram as a density curve.

Thermograms were subsequently stratified by important demographic, laboratory, and comorbidity data to determine whether these covariates influenced differences between SLE patients and controls. Differences between groups were tested for statistical significance by two-way ANOVA with interaction using the thermogram first PC as the response variable. The interaction term was used to determine whether a covariate influenced any differences in thermograms between SLE patients and controls. We also tested for differences in the first PC according to each of the SLE diagnostic criteria (**S1 Table**), serology (Anti dsDNA titer, Anti Ro, Anti La, Anti Smith, ANA titer, and Anti-cardiolipin immunoglobulin G and M), number of ACR criteria and type of SLE onset, patient medications (Prednisone and Hydroxychloroquine), and additional labs (complement C3/C4, hemoglobin, white blood cell / lymphocyte / platelet count, erythrocyte sedimentation rate, globulin, proteinuria, albumin, creatinine, and creatinine clearance) among SLE patients only, to evaluate whether thermogram measures correlated with a certain aspect of SLE or other serological / laboratory data. Serology and other laboratory tests were conducted by the Oklahoma Medical Research Foundation (OMRF) CLIA approved clinical laboratory (for details see ‘Bio-specimen processing and storing’ in Rasmussen et al. [23]). P-values were adjusted for multiple comparisons based on the false-discovery rate (FDR) correction [26].

Last, we evaluated whether the observed differences in thermograms between SLE patients and controls had any diagnostic utility. A modified version of Fisher’s linear discriminant analysis (MLDA) was used to classify subjects as SLE versus control using the information from the thermograms. The MLDA classifier was designed to handle situations where the number of variables (here, excess specific heat capacity at each temperature) potentially exceeds the number of subjects [27]. Determination of SLE was based on the posterior probability of SLE given the thermogram data, as outputted from the MLDA algorithm. Classification based on thermograms alone used a threshold probability of 0.5, while coupling thermogram information together with SLE serological markers used a more stringent threshold of 0.9 (since the goal was to catch cases not detected by the immunological markers).

## Results

### Comparison of thermograms between SLE patients and controls

**Fig 1:**
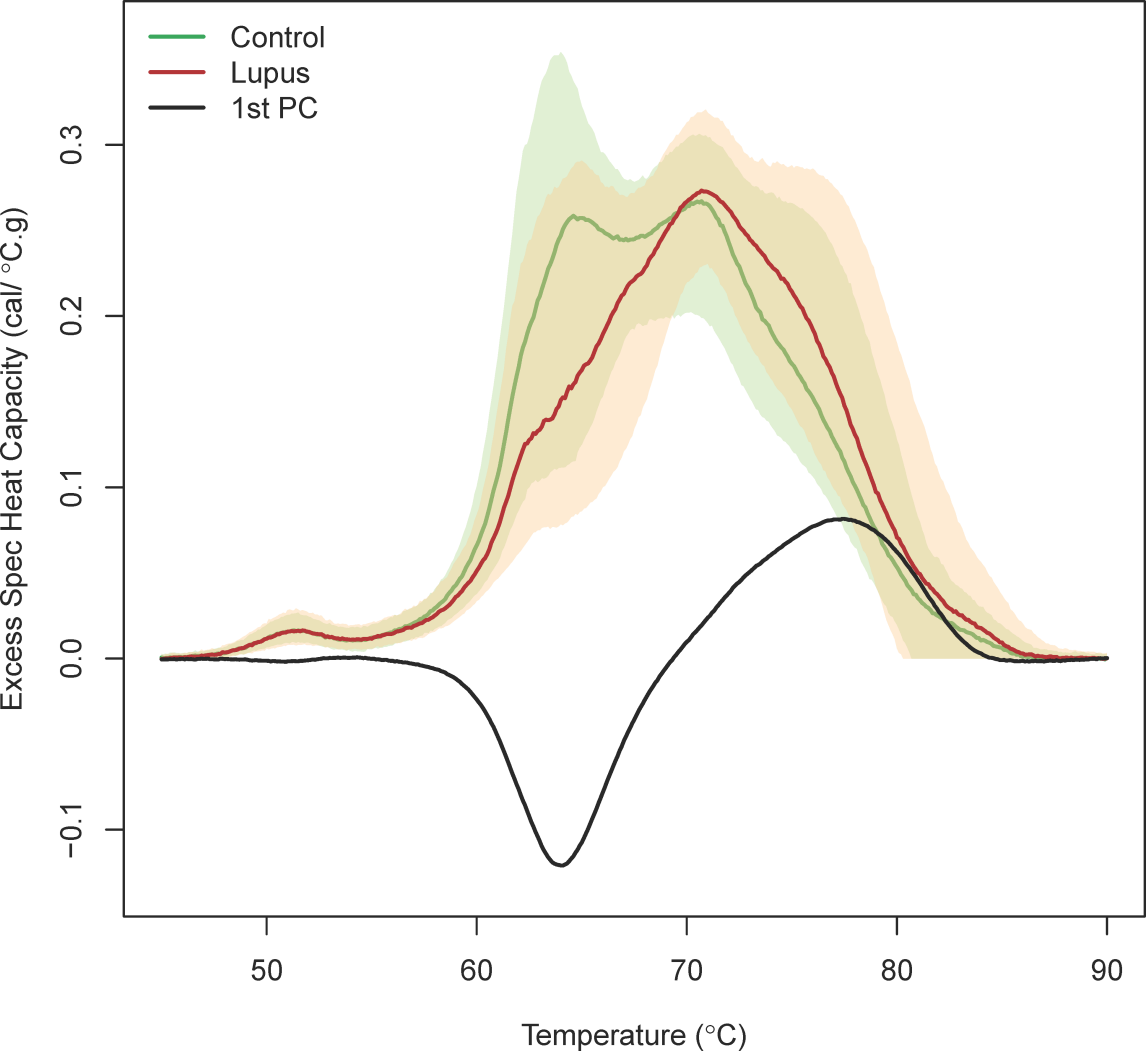
Plot of the median thermogram value at each temperature for lupus and control subjects. Bands represent the 5th and 95th percentiles among subjects at each temperature. The loadings for the first principal component among all subjects are shown as the black line.

A graphical display of the average thermograms separately for SLE patients and controls revealed significant differences between the two sets of subjects (**Fig 1**). In particular, the median thermogram for SLE patients has a markedly reduced initial peak corresponding to ~65°C and the high temperature shoulders of the peak ~70°C is shifted to the right for SLE patients compared with control subjects.

**Fig 2:**
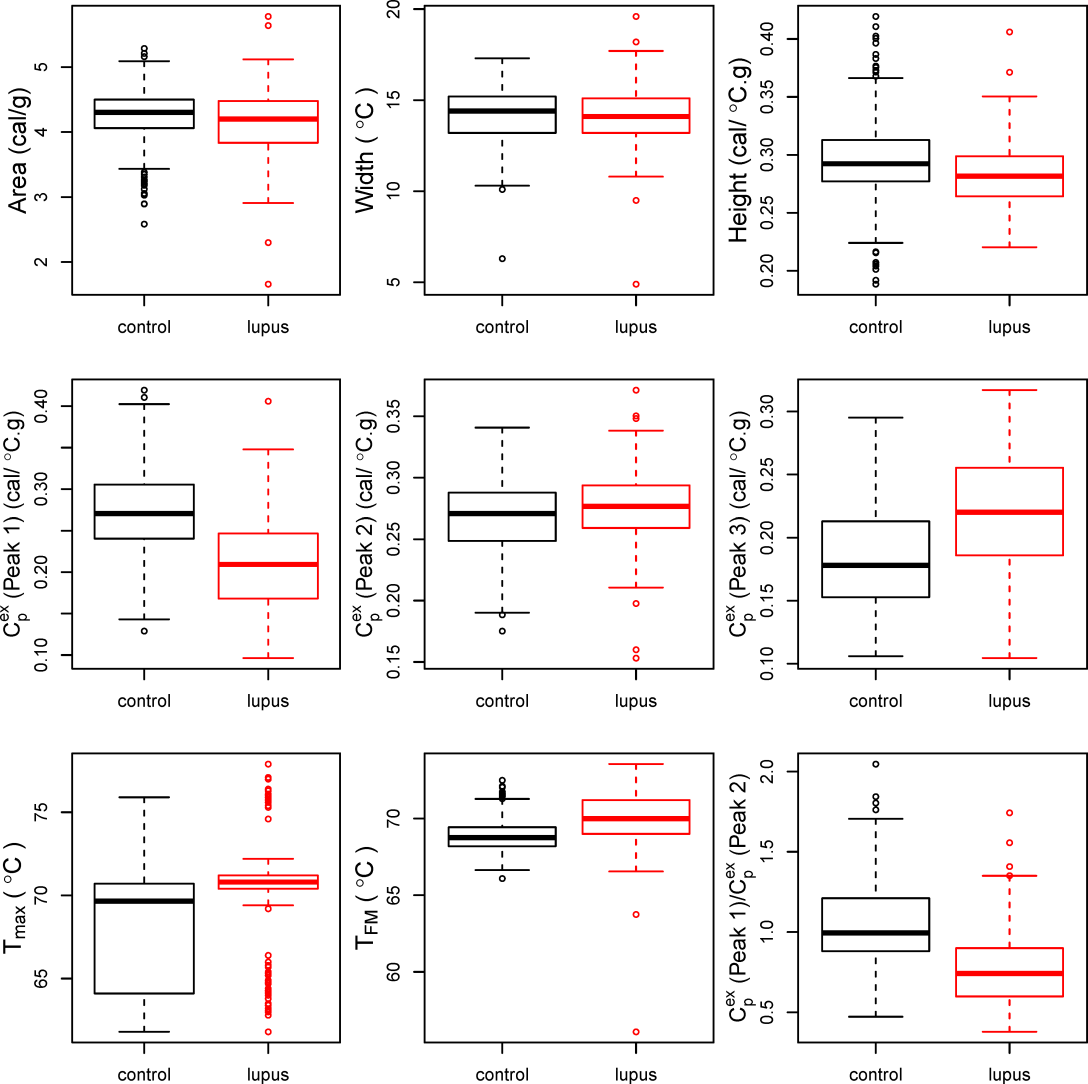
Boxplots of summary statistics calculated for thermograms of lupus patients and controls. Top Row (from left to right): Total area under the curve, width at half height, and height at maximum temperature. Middle Row: Excess specific heat capacity ($C_{p}^{ex}$) at Peak 1 (62–67°C), Peak 2 (69–73°C), and Peak 3 (75–80°C). Bottom Row: Temperature at the maximum peak (T_max_), first moment temperature (T_FM_), and ratio of $C_{p}^{ex}$ at Peak 1 to $C_{p}^{ex}$ at Peak 2.

To examine the ability of thermograms to distinguish SLE from other autoimmune diseases we compared thermograms of SLE patients to controls with autoimmune comorbidities. The thermograms for SLE patients also differed in a similar fashion from controls with osteoarthritis (n = 31 subjects, **S1 Fig**) and rheumatoid arthritis (n = 16 subjects, **S2 Fig**). An overlay of the first principal component (PC) indicates that the loadings for the 1^st^ PC correspond with the shift in the two major peaks seen in the two sets of thermograms. A scree plot for the PCs indicated that six PCs were sufficient to characterize the variability in the thermograms (98.2% of total variability explained, see **S3 Fig**). A multivariate test of differences between SLE and control subjects based on the first six PCs was highly significant (p < 10^−15^), as was the test based on only the first PC (p < 10^−15^). Thermogram summary statistics (as described in the Methods) were calculated and compared between SLE patients and controls (**Fig 2**).

A density plot of T_max_ revealed that there were roughly three prominent peaks among the subjects at 62–67°C, 69–73°C, and 75–80°C (**S4 Fig**). Highly significant differences (p < 0.001, based on the t-test) were present for maximum peak height, T_max_, Peak 1 max $C_{p}^{ex}$, Peak 2 max $C_{p}^{ex}$, the ratio of (Peak 1 max $C_{p}^{ex}$) / (Peak 2 max $C_{p}^{ex}$), and T_FM_. The thermogram peak width at half height was not significantly different between the two groups (p = 0.68), while the total area under the thermogram was slightly higher for controls (p = 0.035). Distinct subpopulations of SLE patients based on differences in T_max_ are observed resulting from variability in the distribution of the thermogram profile. This observation might be related to the clinical status of the patients (for example, active flare versus no flare; with kidney disease versus without kidney disease) and may represent an important application of thermograms for clinical monitoring of these patients.

### Influence of covariates on thermogram differences

Differences between SLE patients and controls along important demographic factors and comorbidities are detailed in **Table 1**.

**Table 1:** Demographics and comorbidities / other conditions by case status.

**Demographics**	**Control** N (%)	**Lupus** N (%)
Gender		
Female	225 (75)	226 (75.3)
Male	75 (25)	74 (24.7)
Ethnicity		
Black	157 (52.3)	159 (53)
White	139 (46.3)	141 (47)
Other	4 (1.3)	0 (0)
Year of birth		
(1924,1944]	70 (23.3)	87 (29)
(1944,1955]	68 (22.7)	78 (26)
(1955,1971]	80 (26.7)	64 (21.3)
(1971,1993]	78 (26)	66 (22)
Missing	4 (1.3)	5 (1.7)
BMI		
[6,16]	1 (0.3)	0 (0)
(16,18.5]	7 (2.3)	13 (4.3)
(18.5,25]	86 (28.7)	89 (29.7)
(25,30]	76 (25.3)	61 (20.3)
(30,71]	77 (25.7)	78 (26)
Missing	53 (17.7)	59 (19.7)
Smoking now		
No	78 (26)	77 (25.7)
Yes	34 (11.3)	41 (13.7)
Not applicable†	132 (44)	108 (36)
Missing	56 (18.7)	74 (24.7)
Number of years smoking		
[0,10]	176 (58.7)	135 (45)
(10,20]	23 (7.7)	28 (9.3)
(20,30]	21 (7)	25 (8.3)
(30,40]	9 (3)	20 (6.7)
(40,50]	5 (1.7)	4 (1.3)
(50,61]	1 (0.3)	2 (0.7)
Missing	65 (21.7)	86 (28.7)
**Comorbidities / Other Conditions**	**Control** N (%)	**Lupus** N (%)
Hypertension		
No	163 (54.3)	121 (40.3)
Yes	84 (28)	159 (53)
Missing	53 (17.7)	20 (6.7)
Arthritis (current or past)		
No	162 (54)	70 (23.3)
Yes	83 (27.7)	209 (69.7)
Missing	55 (18.3)	21 (7)
Osteoarthritis		
No	209 (69.7)	221 (73.7)
Yes	31 (10.3)	46 (15.3)
Missing	60 (20)	33 (11)
Rheumatoid arthritis		
No	226 (75.3)	156 (52)
Yes	16 (5.3)	112 (37.3)
Missing	58 (19.3)	32 (10.7)
Anemia		
No	28 (9.3)	58 (19.3)
Yes	67 (22.3)	156 (52)
Not applicable†	149 (49.7)	45 (15)
Missing	56 (18.7)	41 (13.7)
Hemolytic anemia		
No	81 (27)	165 (55)
Yes	1 (0.3)	14 (4.7)
Not applicable†	149 (49.7)	45 (15)
Missing	69 (23)	76 (25.3)
Leukopenia		
No	63 (21)	63 (21)
Yes	22 (7.3)	138 (46)
Not applicable†	149 (49.7)	44 (14.7)
Missing	66 (22)	55 (18.3)
Thrombocytopenia		
No	71 (23.7)	80 (26.7)
Yes	12 (4)	113 (37.7)
Not applicable†	149 (49.7)	44 (14.7)
Missing	68 (22.7)	63 (21)
Infectious mononucleosis		
No	231 (77)	249 (83)
Yes	15 (5)	25 (8.3)
Missing	54 (18)	26 (8.7)
Psoriasis		
No	230 (76.7)	235 (78.3)
Yes	11 (3.7)	37 (12.3)
Missing	59 (19.7)	28 (9.3)
Scleroderma		
No	242 (80.7)	259 (86.3)
Yes	1 (0.3)	11 (3.7)
Missing	57 (19)	30 (10)
Recurrent chest pain		
No	209 (69.7)	129 (43)
Yes	31 (10.3)	103 (34.3)
Missing	60 (20)	68 (22.7)
Myocardial infarction		
No	235 (78.3)	249 (83)
Yes	8 (2.7)	26 (8.7)
Missing	57 (19)	25 (8.3)
Cancer		
No	232 (77.3)	211 (70.3)
Yes	9 (3)	28 (9.3)
Missing	59 (19.7)	61 (20.3)
Diabetes		
No	214 (71.3)	244 (81.3)
Yes	31 (10.3)	32 (10.7)
Missing	55 (18.3)	24 (8)

†Per the LFRR database manual, ‘Not applicable’ means ‘Not applicable, question does not apply, no answer required’

**Fig 3:**
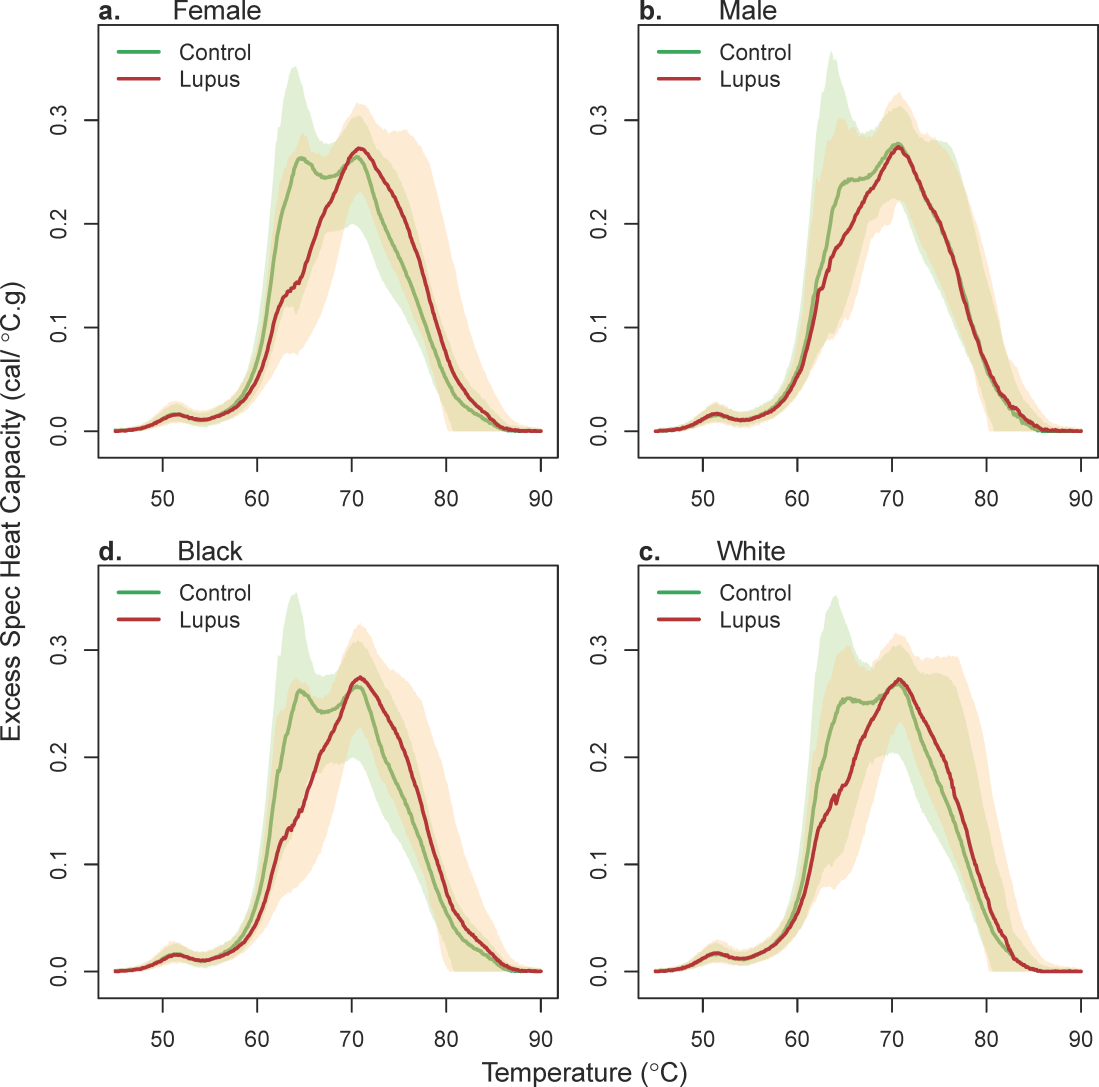
Plot of the median thermogram value at each temperature for lupus and control subjects stratified by gender and ethnicity. Bands represent the 5th and 95th percentiles among subjects at each temperature.

Demographic factors were similar between the two groups, while expectedly conditions associated with SLE differed. These covariates were subsequently evaluated to determine whether they were effect modifiers for the differences in thermograms between SLE patients and controls, by investigating the significance of the interaction term between the covariate and case / control status in a linear model with the first PC of the thermograms as the response. Of the 22 variables listed in **Table 1**, only sex and anemia were found to have a statistically significant interaction with case / control status after adjusting for multiple comparisons (FDR adjusted p < 0.001 and 0.02, respectively; see **S2 Table**). Ethnicity had a significant unadjusted p-value (p = 0.04), but was not significant after accounting for multiple comparisons. The interaction can be demonstrated visually by viewing average thermogram profiles for SLE and control subjects stratified by sex and ethnicity (**Fig 3**) and anemia (**S5 Fig**). Separation between SLE and control subjects is more evident for females, black ethnicity, and subjects with anemia.

In a similar fashion, we investigated whether variations in thermograms among SLE patients were associated with any of the SLE diagnostic criteria and additional laboratory data (**S3 Table**). The most significant result was for anti-cardiolipin immunoglobulin G (IgG, FDR adjusted p = 0.10). Patients with higher IgG values had thermograms shifted to the left, which was true for both SLE patients and controls (**S6 Fig**).

### Classification of SLE patients versus controls

**Fig 4:**
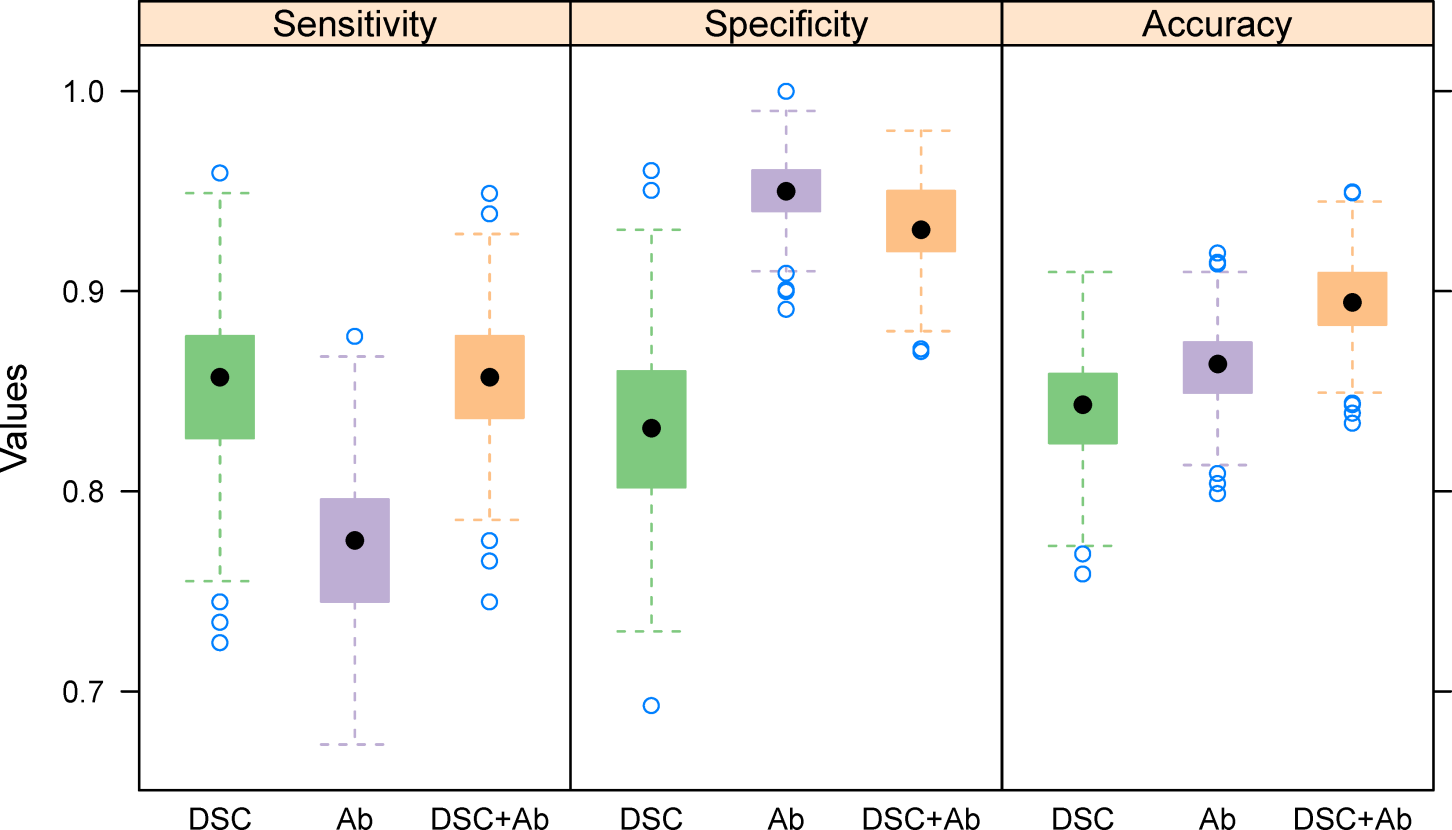
Sensitivity, specificity, and overall accuracy for classifying lupus patients vs. controls based on DSC thermograms only (DSC), antibody tests only (Ab), and combined DSC / antibody tests (DSC+Ab). Boxplots represent values from 1000 test data sets created by splitting the data randomly into training (two thirds) and testing (one third) sets.

To determine whether the observed differences in thermograms between SLE patients and controls had any diagnostic utility, we used the MLDA [27] program to classify subjects as SLE versus control based on the information from the thermograms. We compared three different diagnostic models: a) a model based on DSC thermograms only (DSC), b) a model based on antibody tests only (Ab), and c) a model based on coupling the antibody test with thermograms (DSC+Ab). For purposes of establishing a biomarker based comparator we selected one which had optimal performance (accuracy) in our data. That is, we have classified a subject as SLE positive if any of the Anti dsDNA titer, Anti Ro, Anti La, Anti Smith, and ANA titer tests were positive. Specifically, a titer of 1:30 or higher was considered positive for the Anti dsDNA test, while a value of 1:360 or higher was considered positive for the ANA titer. The high value for the ANA titer was used to achieve optimal overall accuracy (otherwise, sensitivity would be 100% but specificity would be much lower). All other lab values were simply reported as positive or negative in the LFRR database. All of the antibody tests are highly specific for SLE but have low sensitivity, so combining them in this fashion produced the optimal test based on antibodies alone. Data were randomly split 1000 times into training (two thirds) and test (one third) data sets, and sensitivity, specificity, and overall accuracy were calculated for each model for each split. Median sensitivity, specificity, and overall accuracy based on classification using plasma thermograms was 86%, 83%, and 84% compared to 78%, 95%, and 86% based on the combined serological markers (**Fig 4**). To combine the models and improve the sensitivity of the antibody based test while maintaining the same specificity, we classified a subject as SLE positive if either the antibody test was positive *or* the predicted probability of SLE based on the thermogram passed a high threshold (probability > 0.9). Results indicate that the sensitivity (86%) and overall accuracy (89%) were improved relative to the antibody only test, with only a small drop in specificity (93%) (**Fig 4**).

We also investigated the classification performance of our DSC-based and combined DSC+Ab model on specific subsets of patients. We chose two comparator groups, rheumatoid arthritis and osteoarthritis to determine the effect of inflammatory disease on the thermogram profile. Rheumatoid arthritis was chosen as an autoimmune disease that is usually limited in its target involvement but can be associated with a robust systemic inflammatory response. Osteoarthritis was chosen because it represents a low grade inflammatory process. **S7 and S8 Figs** show that comparable results were obtained when classifying SLE patients versus controls with a comorbidity of either osteoarthritis or rheumatoid arthritis, with median overall accuracies based on the DSC profiles of 85% and 86%, respectively. Median accuracy and inter-quartile range (IQR) for the DSC and antibody based models stratified by gender and ethnicity are given in **Table 2**. DSC accuracy was lower among white males compared to the other three sex / ethnic groups. However, in all cases combining DSC and antibody information improved the overall accuracy relative to the Ab only models by increasing the overall sensitivity. This increase was most pronounced in white females, where sensitivity improved from 68% in the Ab only model to 80% in the DSC+Ab model and overall accuracy improved from 79% to 84%. In most cases (the exception being white males), the specificity of the combined DSC+Ab model was only slightly impacted (1% decrease or less) relative to the Ab only model.

**Table 2:** Accuracy of DSC, antibody only, and combined antibody + DSC classifiers in patient subsets according to race and gender.

**DSC**				
	Black Females	Black Males	White Females	White Males
Sensitivity	0.89	0.86	0.86	0.75
	(0.86, 0.92)	(0.75, 0.91)	(0.82, 0.90)	(0.68, 0.83)
Specificity	0.84	0.88	0.87	0.73
	(0.80, 0.87)	(0.80, 1.00)	(0.83, 0.91)	(0.65, 0.80)
Accuracy	0.86	0.86	0.87	0.74
	(0.84, 0.89)	(0.80, 0.91)	(0.84, 0.89)	(0.69, 0.79)
**Antibody Only**				
	Black Females	Black Males	White Females	White Males
Sensitivity	0.87	0.78	0.68	0.69
	(0.84, 0.90)	(0.67, 0.86)	(0.63, 0.72)	(0.62, 0.75)
Specificity	0.97	1.00	0.90	1.00
	(0.95, 0.98)	(0.90, 1.00)	(0.87, 0.93)	(0.95, 1.00)
Accuracy	0.92	0.87	0.79	0.84
	(0.90, 0.94)	(0.81, 0.91)	(0.76, 0.82)	(0.80, 0.87)
**Antibody + DSC**				
	Black Females	Black Males	White Females	White Males
Sensitivity	0.93	0.85	0.80	0.78
	(0.91, 0.95)	(0.75, 0.90)	(0.75, 0.85)	(0.72, 0.83)
Specificity	0.96	1.00	0.89	0.94
	(0.93, 0.98)	(0.90, 1.00)	(0.86, 0.92)	(0.92, 1.00)
Accuracy	0.94	0.89	0.84	0.86
	(0.93, 0.96)	(0.85, 0.94)	(0.82, 0.87)	(0.83, 0.90)

Entries in each cell are median and inter-quartile range (IQR, 25^th^ percentile and 75^th^ percentile).

## Discussion

DSC analysis of biofluid samples is an emerging area of proteomics research with demonstration of preliminary utility for the discrimination of disease subjects from controls in multiple disease types. We have previously published a pilot study showing substantial changes in thermograms for SLE patients compared to healthy controls. To further investigate these observations, we embarked upon a much larger study to confirm the utility of DSC for discrimination of SLE from controls and to evaluate additional demographic and clinical factors of interest. Further, this study is the first to demonstrate how thermograms can be used to improve upon an existing serological based classification, here by increasing both sensitivity and overall accuracy for SLE patients versus controls. This gives a template for developing thermograms as a potential complementary diagnostic tool.

Application of the MLDA approach to thermogram data determined median diagnostic sensitivity, specificity and overall accuracy among the 1000 test sets of 86%, 83% and 84%, respectively, for the classification of SLE patients versus non SLE controls. These results compare well to the study by Fish et al. [22] and Garbett and Brock [28] where median overall accuracy ranged from 74–88%. Further, by including information from thermograms the median sensitivity of our defined antibody test for SLE was improved from 78% to 86% and the overall accuracy improved from 86% to 89%, while the specificity was minimally impacted (reduced from 95% to 93%). The classification cut-offs for the thermogram data were based on posterior probabilities as determined by the MLDA algorithm, and a noted limitation of the current approach is the difficulty in interpreting the resulting thermogram ‘signature’ for separating cases and controls (c.f. Fig 7 in Garbett and Brock [28]). Future research will address ways to enhance this.

The ability of the thermograms to accurately classify SLE patients varied according to several demographic factors (sex, ethnicity) and other health conditions (anemia), with highest accuracy in females and black subjects. In our previous study of 100 healthy plasma samples [7] we also found differences in thermograms according to sex and ethnicity, and these variations can form the basis for development of specific control populations. However, in every case the overall accuracy of the thermogram based models was comparable to the optimal antibody based test and the combined antibody / thermogram models improved the sensitivity and overall accuracy of the antibody tests. No other statistically significant demographic / health-related factors were identified which impacted the thermogram differences between SLE patients and controls. That is, similar differences in thermograms were observed between SLE and control subjects with high blood pressure, arthritis, infectious mononucleosis, recurrent chest pain, diabetes, and cancer (c.f. **S2 Table**). However, in some of these cases (e.g., cancer) the number of subjects with the condition is too small to make definitive conclusions. And while we determined that thermogram profiles for SLE patients differed from controls with osteoarthritis and rheumatoid arthritis, we were unable to investigate whether SLE patient profiles differed from other important connective tissue diseases such as primary Sjogren’s syndrome (n = 4 patients in our data). Lastly, patient medications (prednisone and hydroxychloroquine) did not influence thermogram changes resulting from SLE status, although this would require additional testing.

Use of plasma samples from the LFRR is extremely valuable in exploring the potential of DSC analysis for detection of SLE but has limitations which prevent the full diagnostic and prognostic utility of DSC profiling from being observed. First, a patient’s current disease status (e.g., different organ involvement, disease remission vs. flare) and measures of disease activity, such as BILAG, SLAM and SLEDAI (see, e.g., Romero-Diaz et al. [29]), at the time of blood sample collection are not recorded in the database, and this can impact the thermogram. Temporal variation may be observed in thermograms which can potentially be correlated with changes in the physiological state of the disease. Evaluating how thermogram changes track with disease severity over time are important for determining the full clinical applicability of DSC profiling. Second, it is important to include well-defined control groups to examine the influence on thermogram profile of common SLE comorbidities and other diseases. These groups should include appropriate comparisons of active and inactive inflammatory disease with active and inactive SLE. Third, the determination of SLE in the LFRR database is based on the ACR criteria [24], and the revised SLICC classification criteria may have increased sensitivity for SLE [30, 31]. Finally, on a technical note, this study involved the analysis of plasma specimens banked over a number of years by the LFRR. It was not possible to match storage times of the 600 samples examined in this study. We have previously examined the effect of a number of pre-analytical variables on thermogram profiles, including different storage times, temperatures and freeze-thaw cycles [25]. We did not find any effect on thermogram profile of storage at -20°C or -80°C for times of one week to six months. However, we have not examined the effect of long-term storage of thermograms, which will be examined in future studies. However, despite the limitations of the LFRR data, thermogram classification is comparable to antibody based testing, completed at the time of specimen collection before banking in the biorepository, and overall accuracy is improved when thermogram and antibody based testing are combined.

The comorbidity data in the LFRR were obtained via self-reported questionnaires and were not confirmed by physician information or testing. This may result in an over or under-reporting of certain conditions, for instance the prevalence of rheumatoid arthritis in our SLE patient group which was unusually high. Since a majority of SLE subjects do experience arthritis, this could reflect a misunderstanding on the caregiver or patient’s part. However, to address concerns about the representativeness of our sample to the general lupus population we have compared the percentage of lupus subjects in our study satisfying the 11 ACR criteria with previous data from both national and international studies [32]. In particular, we compared our percentages to those presented for the 1982 revised criteria for the classification of SLE lupus [33]. Compared to that study, our SLE patient population had similar percentages of discoid rash (18% to 18.3% in our sample), photosensitivity (43% to 47% in our sample), oral ulcers (27% to 30% in our sample), Arthritis (86% to 83% in our sample), hematologic disorder (59% to 67% in our sample), immunologic disorder (85% to 82% in our sample), and antinuclear antibody (99% to 100% in our sample). Our SLE patient group did have lower prevalence of malar rash (43.3% vs. 57% in 1982 data), serositis (40% vs. 56% in 1982 data), renal disorder (37% vs. 51% in 1982 data) and neurological disorder (12% vs. 20% in 1982 data). However, in each of these cases the prevalence of the criteria in our study was well within the percentage range reported for the four European studies in Cerovec et al. [32] (c.f. Table 2 in that paper, which compares data from a Croatian sample to studies done in the USA, Europe, Germany and Norway). Hence, based on these data we feel confident that our SLE patient sample is by and large representative of the general SLE patient population, although we note again that the mission of the LFRR repository is to progress SLE genetics research and thus has a large percentage of samples collected from multiplex pedigrees.

We investigated a host of SLE phenotypes, serology, and laboratory measurements to uncover potential biophysical underpinnings of thermograms differences between SLE patients and controls. Our most prominent association was somewhat counter-intuitive, in that patients with *lower* anti-cardiolipin IgG and IgM levels had thermograms with a more prominent transition around 75–80°C, a region of the thermogram we have previously described as dominated by immunoglobulin transitions [6]. The same observation was also noted among controls. None of the other candidates we investigated (including C3/C4 complements, ANA titers, etc.) were significantly associated with thermogram shifts. We did see a stronger separation between SLE patients and controls among those with anemia. One potential explanation for this finding is the expected association between anemia and thrombocytopenia in SLE, where the latter is usually a marker of more severe disease. However, thrombocytopenia alone was not associated with thermogram shifts and these observations require further investigation. Although a clear association between thermogram changes and specific SLE factors was not found this was a valuable first step in exploring the mechanism of thermogram modulation in SLE which warrants further study. Thermogram changes are reflective of changes in the thermal properties of the major plasma proteins which could be related to differences in structural stability or interacting networks of these proteins. The basis of these changes is currently being examined in our lab and has the potential to reveal new aspects of SLE biology.

One could envision several ways to apply DSC in a clinical setting. First, DSC could be used as another measure to confirm a case of SLE when antibody tests are all negative but other clinical symptoms are present. In this case using DSC with a high threshold probability is warranted to maintain high specificity for SLE. Alternatively, DSC could be considered as a single test alternative to the suite of antibody tests, based on the overall sensitivity and specificity of DSC alone for SLE. In this case, a lower threshold probability is needed for SLE to ensure a high enough sensitivity. Lastly, DSC could be applied primarily for detection within certain demographic groups where antibody-based tests are less effective (e.g., white females based on our results). There is also an unmet need for early SLE diagnosis, particularly for cases presenting with <4 ACR criteria but with major organ disease, as well as for SLE surveillance, particularly for early detection of changes in disease activity, organ involvement or therapeutic response. Testing DSC in such cases would be interesting, though a prospective study would be required. In summary, while a thorough evaluation of DSC in a clinical setting is needed to confirm the utility of the biomarker, this report provides an important initial step to establish its potential and lays the groundwork for future studies.

## Supporting information

S1 Table SLE criteria evaluated in the study. (DOCX)

S2 Table P-values for interaction between covariate and case / control status in a statistical model with the first PC of the thermograms as the response variable. (DOCX)

S3 Table P-values for association between the first PC of the thermograms and ACR diagnostic criteria listed in S1 Table among SLE patients. (DOCX)

S4 Table DSC thermogram data for all patient samples included in this study. This file contains the DSC data from all 592 patients (298 lupus, 294 control) included in this study. The data has dimension 266,992 rows by 4 columns. Each column contains a unique variable while each row contains DSC data for a given subject at a given temperature. The data is in ‘long’ format so that data for a single subject spans multiple rows. The following is a description of the variables:
**Subject.ID**: A unique identifier for each subject
**SLE.status**: Disease status of the patient, either ‘lupus’ or ‘control’
**Temperature**: Temperature (in degrees Celsius)
**DSC**: DSC (thermogram) value in cal/°C.g
Clinical information can be requested from the Lupus Family Registry and Repository / Oklahoma Rheumatic Disease Research Cores Center at https://omrf.org/research-faculty/core-facilities/ordrcc/ or through contacting the Oklahoma Medical Research Foundation.
(CSV)

S1 Fig Plot of the median thermogram value at each temperature for lupus and osteoarthritis patients. Bands represent the 5th and 95th percentiles among subjects at each temperature. The loadings for the first principal component among all subjects are shown as the black line.
(TIF)

S2 Fig Plot of the median thermogram value at each temperature for lupus and rheumatoid arthritis patients. Bands represent the 5th and 95th percentiles among subjects at each temperature. The loadings for the first principal component among all subjects are shown as the black line.
(TIF)

S3 Fig Scree plot for principal components of DSC thermograms based on all subjects (lupus patients and controls). (TIF)

S4 Fig Density of temperature at maximum peak thermogram height (Tmax) for controls and lupus patients. The density plots reveal roughly three prominent peaks among the subjects at 62–67°C, 69–73°C, and 75–80°C (the latter being present only among lupus patients).
(TIF)

S5 Fig Plot of the median thermogram value at each temperature for lupus and control subjects stratified by presence / absence of anemia. Not applicable indicates that the study question did not apply. Bands represent the 5th and 95th percentiles among subjects at each temperature.
(TIF)

S6 Fig Plot of the median thermogram value at each temperature for lupus and control patients stratified by level of anti-cardiolipin immunoglobulin G. Cut-point at the median value of 6). Bands represent the 5th and 95th percentiles among subjects at each temperature.
(TIF)

S7 Fig Sensitivity, specificity, and overall accuracy for classifying lupus patients vs. osteoarthritis patients based on DSC thermograms only (DSC), antibody tests only (Ab), and combined DSC / antibody tests (DSC+Ab). Boxplots represent values from 1000 test data sets created by splitting the data randomly into training (two thirds) and testing (one third) sets.
(TIF)

S8 Fig Sensitivity, specificity, and overall accuracy for classifying lupus patients vs. rheumatoid arthritis patients based on DSC thermograms only (DSC), antibody tests only (Ab), and combined DSC / antibody tests (DSC+Ab). Boxplots represent values from 1000 test data sets created by splitting the data randomly into training (two thirds) and testing (one third) sets.
(TIF)

## Abbreviations

DSC: differential scanning calorimetry
SLE: systemic lupus erythematosus
MLDA: modified linear discriminant analysis
LFRR: Lupus Family Registry and Repository
OMRF: Oklahoma Medical Research Foundation
PC: principal component
FDR: false discovery rate
IQR: inter-quartile range
