# Supplementary material for: Characterization and classification of lupus patients based on plasma thermograms
Source: PLoS One. 2017 Nov 17;12(11):e0186398. doi: 10.1371/journal.pone.0186398 (PMC5693473; doi:10.1371/journal.pone.0186398)
Supplement: S1 Table — (DOCX) [file pone.0186398.s001.docx]

**S1 Table. SLE criteria evaluated in the study.**

| Criterion **^1^** | Description | N (%) ^2^ |
| --- | --- | --- |
| **Serological ACR criteria** |  |  |
| 1. Immunological disorder | Positive Anti-dsDNA, Anti-Smith, or antiphospholipid test (details below) | 246 (82.0%) |
| Anti-dsDNA | Autoantibodies to native double-stranded DNA. High specificity for SLE but low sensitivity. |  |
| Anti-Smith | Autoantibodies to Smith nuclear antigen. High specificity for SLE but low sensitivity. |  |
| Anti-cardiolipin IgG | Autoantibodies to cardiolipin, a mitochondrial membrane phospholipid, resulting in thrombosis. |  |
| Lupus anticoagulant | Autoantibodies that bind cell membrane phospholipids and proteins. Name derived from in vitro anticoagulant properties but in vivo interaction with platelet membrane phospholipids results in platelet aggregation and prothrombotic effects. |  |
| False +VDRL | A false positive venereal disease research laboratory (VDRL) test for at least 6 months confirmed by a Treponema pallidum immobilization or fluorescent treponemal antibody absorption test |  |
| 2. ANA titer | Autoantibodies to nuclear as well as cytoplasmic cell components. High sensitivity for autoimmune diagnosis but low diagnostic specificity for SLE. | 300 (100%) |
| 3. Renal disorder | Either proteinuria or presence of cellular casts (details below) | 113 (37.7%) |
| Proteinuria | Abnormal levels of protein in the urine indicating kidney dysfunction |  |
| Cellular casts | Cylindrical structures typically of red or white blood cells produced by the kidney and excreted into the urine. Indicates glomerular damage, inflammation or infection. |  |
| 4. Hematologic disorder | Presence of one of hemolytic anemia, leukopenia, lyphopenia, or thrombocytopenia (details below) | 201 (67.0%) |
| Hemolytic anemia | Decreased red blood cell count as a result of autoantibody-mediated destruction. Common in about half of SLE patients. |  |
| Leukopenia | Decreased white blood cell count as a result of autoantibody-mediated destruction and indicative of an increased risk of infection. |  |
| Lyphopenia | Aka Lymphopenia, decreased levels of lymphocytes in the blood. Present in about 75% of SLE patients [[1](#_ENREF_1)]. |  |
| Thrombocytopenia | Decreased platelet counts resulting from immune-mediated destruction or drug-impaired production. Mild thrombocytopenia observed in a quarter to a half of SLE patients. |  |
| **Clinical ACR criteria** |  |  |
| 5. Malar rash | Rash localized on the nose and cheekbone (butterfly rash) seen in about half of SLE patients. | 133 (43.3%) |
| 6. Discoid rash | Raised scaly rash on the head, arms, chest or back observed in about a quarter of SLE patients. | 55 (18.3%) |
| 7. Photosensitivity | Skin rash as a result of unusual reaction to sunlight. | 141 (47.0%) |
| 8. Oral ulcers | Mouth ulceration that is usually painless. | 91 (30.3%) |
| 9. Arthritis | Tenderness and swelling of peripheral joints, usually hand and wrist. | 250 (83.3%) |
| 10. Serositis | Inflammation of serous tissues, typically the lungs (pleuritis) and heart (pericarditis). | 121 (40.3%) |
| 11. Neurologic disorder | Neurological symptoms including headaches, seizures and psychosis resulting from damage to the central or peripheral nervous systems. | 36 (12.0%) |

**^1^** ACR criteria were scored on an integer scale from 0 to 3 based on increasing level of evidence that the clinical symptom is present. Subscales (e.g., 2A, 2B, 2C for Arthritis and Renal Disorder and 3A for Hematological and Immunological Disorders) are present for certain criteria. For each criteria, the variable corresponding to the highest value from medical records, patient interview, or other interview was used. A positive diagnosis of lupus is based on the presence of four of the eleven criteria. For details see Rassmussen *et al.* [[2](#_ENREF_2)] and the references therein.

**^2^** Number and percent having convincing evidence (ACR criteria integer score = 3) that the criteria is met

**References**

1. Rivero SJ, Díaz-Jouanen E, Alarcón-Segovia D. Lymphopenia in systemic lupus erythematosus. Clinical, diagnostic, and prognostic significance. Arthritis Rheum. 1978;21(3):295-305.

2. Rasmussen A, Sevier S, Kelly JA, Glenn SB, Aberle T, Cooney CM, et al. The lupus family registry and repository. Rheumatology. 2011;50(1):47-59.
