# Supplementary material for: Characterization and classification of lupus patients based on plasma thermograms
Source: PLoS One. 2017 Nov 17;12(11):e0186398. doi: 10.1371/journal.pone.0186398 (PMC5693473; doi:10.1371/journal.pone.0186398)
Supplement: S2 Table — (DOCX) [file pone.0186398.s002.docx]

**S2 Table. P-values for interaction between covariate and case / control status in a statistical model with the first PC of the thermograms as the response variable.**

| **Demographics** | Unadjusted p-value | FDR^1^ adjusted p-value |
| --- | --- | --- |
| Gender | <0.001 | <0.001 |
| Ethnicity | 0.04 | 0.31 |
| Year of birth | 0.96 | 0.97 |
| BMI | 0.48 | 0.77 |
| Smoking now | 0.87 | 0.97 |
| Number of years smoking | 0.39 | 0.77 |
| **Comorbidities / Other Conditions** |  |  |
| High blood pressure | 0.08 | 0.40 |
| Arthritis (current or past) | 0.51 | 0.77 |
| Osteoarthritis | 0.68 | 0.91 |
| Rheumatoid arthritis | 0.29 | 0.70 |
| Anemia | 0.002 | 0.02 |
| Hemolytic anemia | 0.06 | 0.36 |
| Leukopenia | 0.19 | 0.62 |
| Thrombocytopenia | 0.16 | 0.62 |
| Infectious mononucleosis | 0.46 | 0.77 |
| Psoriasis | 0.95 | 0.97 |
| Scleroderma | 0.39 | 0.77 |
| Recurrent chest pain | 0.56 | 0.78 |
| Myocardial infarction | 0.92 | 0.97 |
| Cancer | 0.87 | 0.97 |
| Diabetes | 0.21 | 0.62 |

^1^ FDR = False Discovery Rate
