# Supplementary material for: Characterization and classification of lupus patients based on plasma thermograms
Source: PLoS One. 2017 Nov 17;12(11):e0186398. doi: 10.1371/journal.pone.0186398 (PMC5693473; doi:10.1371/journal.pone.0186398)
Supplement: S3 Table — (DOCX) [file pone.0186398.s003.docx]

**S3 Table. P-values for association between the first PC of the thermograms and ACR diagnostic criteria listed in Supplementary Table 1 among SLE patients.**

|  | Unadjusted p-value | FDR^1^ adjusted p-value |
| --- | --- | --- |
| **Serological ACR criteria** ^2^ |  |  |
| 1. Immunological disorder (highest)^3^ | 0.42 | 0.70 |
| Anti-dsDNA (OMRF serology) ^4^ | 0.19 | 0.60 |
| Anti-Smith (OMRF serology) | 0.37 | 0.68 |
| Antiphospholipid Ab ^5^ (highest) | 0.016 | 0.28 |
| Anti-cardiolipin IgG (OMRF serology) | 0.29 | 0.67 |
| Lupus anticoagulant (med record) ^6^ | 0.10 | 0.53 |
| False +VDRL (med record) | 0.43 | 0.71 |
| 2. ANA titer (highest) ^7^ | -- | -- |
| 3. Renal disorder (highest) | 0.60 | 0.80 |
| Proteinuria (med record) | 0.21 | 0.60 |
| Cellular casts (med record) | 0.60 | 0.80 |
| 4. Hematologic disorder (highest) | 0.79 | 0.91 |
| Hemolytic anemia (med record) | 0.10 | 0.53 |
| Leukopenia (med record) | 0.89 | 0.94 |
| Lyphopenia (med record) | 0.16 | 0.60 |
| Thrombocytopenia (med record) | 0.71 | 0.86 |
| **Clinical ACR criteria** |  |  |
| 5. Malar rash (highest) | 0.08 | 0.50 |
| 6. Discoid rash (highest) | 0.18 | 0.60 |
| 7. Photosensitivity (highest) | 0.98 | 1.00 |
| 8. Oral ulcers (highest) | 0.06 | 0.42 |
| 9. Arthritis (highest) | 0.30 | 0.67 |
| 10. Serositis (highest) | 0.22 | 0.60 |
| Pericarditis (med record) | 0.81 | 0.92 |
| Pleuritis (med record) | 0.03 | 0.35 |
| 11. Neurologic disorder (highest) | 0.05 | 0.39 |
| Seizures (med record) | 0.14 | 0.56 |
| Psychosis (med record) | 0.13 | 0.56 |
| **Other SLE related criteria** |  |  |
| Number ACR criteria (med records and OMRF serology) | 0.13 | 0.56 |
| Type SLE onset (acute, insidious, or indeterminate) | 0.28 | 0.67 |
| Additional autoimmune illness (y/n) | 0.64 | 0.80 |
| **OMRF antibody laboratory testing** |  |  |
| Anti-dsDNA titer (highest test value documented) | 0.31 | 0.67 |
| Anti-Smith (positive or negative) | 0.33 | 0.67 |
| Anti-Ro (positive or negative) | 0.55 | 0.79 |
| Anti-La (positive or negative) | 0.98 | 1.00 |
| ANA titer (highest test value documented) | 0.46 | 0.73 |
| Anti-cardiolipin immunoglobulin G (highest test value documented) | 0.002 | 0.10 |
| Anti-cardiolipin immunoglobulin M (highest test value documented) | 0.007 | 0.19 |
| **Additional laboratory testing** |  |  |
| Complement C3 (lowest test value documented) | 0.43 | 0.68 |
| Complement C4 (lowest test value documented) | 0.19 | 0.55 |
| Hemoglobin (lowest test value documented) | 0.62 | 0.80 |
| White blood cell (lowest test value documented) | 0.29 | 0.60 |
| Lymphocyte count (lowest test value documented) | 0.29 | 0.60 |
| Platelet count (lowest test value documented) | 0.88 | 0.94 |
| Erythrocyte sedimentation rate (highest test value documented) | 0.87 | 0.94 |
| Globulin (highest test value documented) | 0.12 | 0.52 |
| Proteinuria measured in mg/24 hours (highest test value documented) | 0.54 | 0.77 |
| Albumin (lowest test value documented) | 0.65 | 0.80 |
| Creatinine (highest test value documented) | 0.52 | 0.77 |
| Creatinine clearance (lowest test value documented) | 0.74 | 0.88 |
| **Patient medications** |  |  |
| Prednisone (Deltasone, Meticorten) | 0.40 | 0.68 |
| Hydroxychloroquine (Plaquenil) | 0.04 | 0.39 |

^1^ FDR = False Discovery Rate

^2^ Tests of association here are based on an ACR criteria evidence score (0-3). These values may differ from the ‘**OMRF antibody laboratory testing**’ section where in that section they are based on the reported laboratory result (either a titer value or positive / negative determination).

^3^ Highest = Maximum score between the medical records, subject interview, or other medically convincing source

^4^ OMRF serology = Based on OMRF serological testing [[1](#_ENREF_1)]

^5^ Summary of antiphospholipid antibody tests showing highest score of false +VDRL, lupus anticoagulant, and anticardiolipin antibody documented via subject interview, physician interview, medical records, or other medically convincing source

^6^ Med record = Based on information in the medical record

^7^ No test could be done for ACR criteria of ANA titer since all lupus patients were found to have convincing evidence of this. In contrast a test for association with actual ANA titer values (under ‘**OMRF antibody laboratory testing**’) is possible.

**References**

1. Rasmussen A, Sevier S, Kelly JA, Glenn SB, Aberle T, Cooney CM, et al. The lupus family registry and repository. Rheumatology. 2011;50:47-59.
