# Supplementary figures and images for: Characterization and classification of lupus patients based on plasma thermograms
Source: PLoS One. 2017 Nov 17;12(11):e0186398. doi: 10.1371/journal.pone.0186398 (PMC5693473; doi:10.1371/journal.pone.0186398)

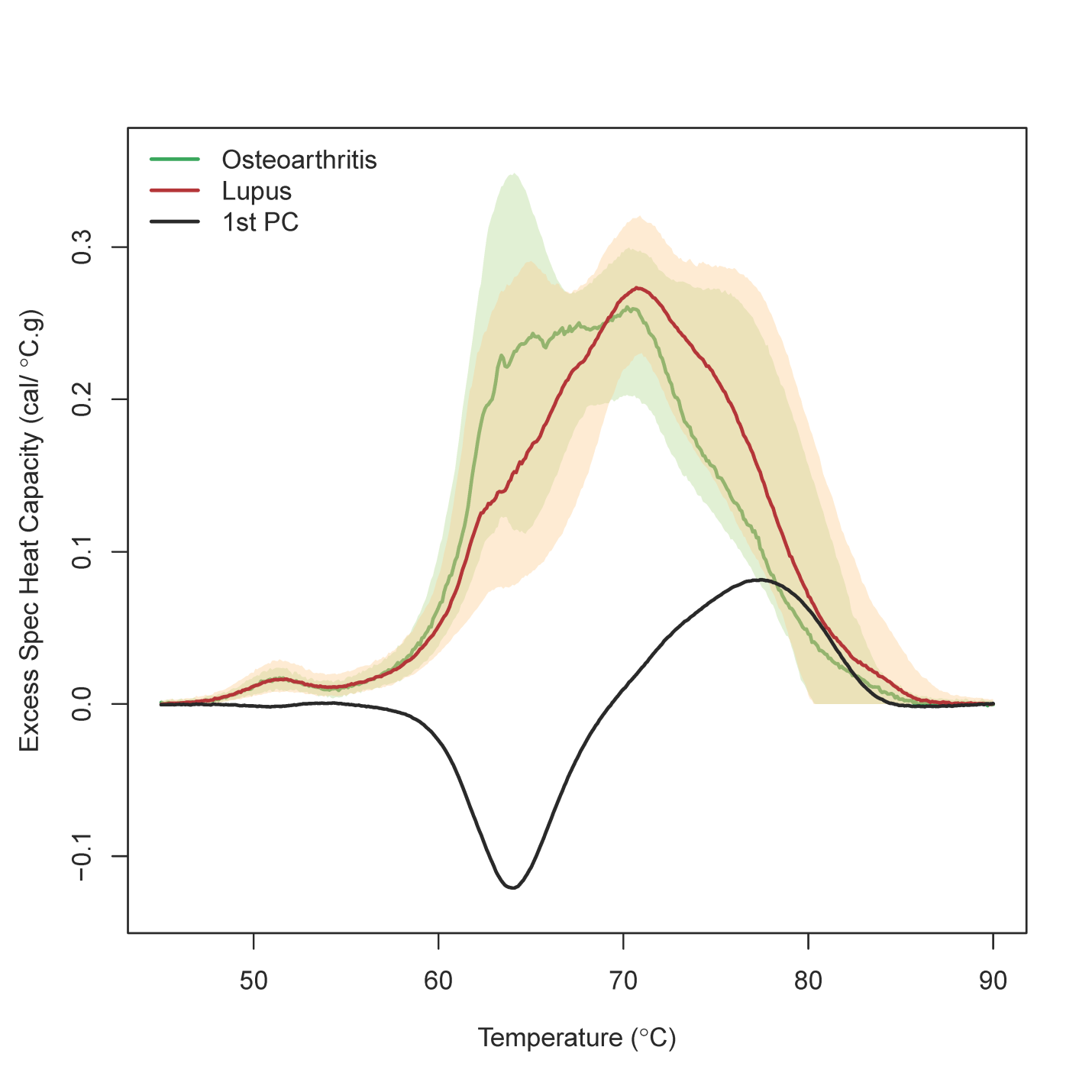

Supplement: S1 Fig — Bands represent the 5th and 95th percentiles among subjects at each temperature. The loadings for the first principal component among all subjects are shown as the black line. (TIF) [file pone.0186398.s005.tif]

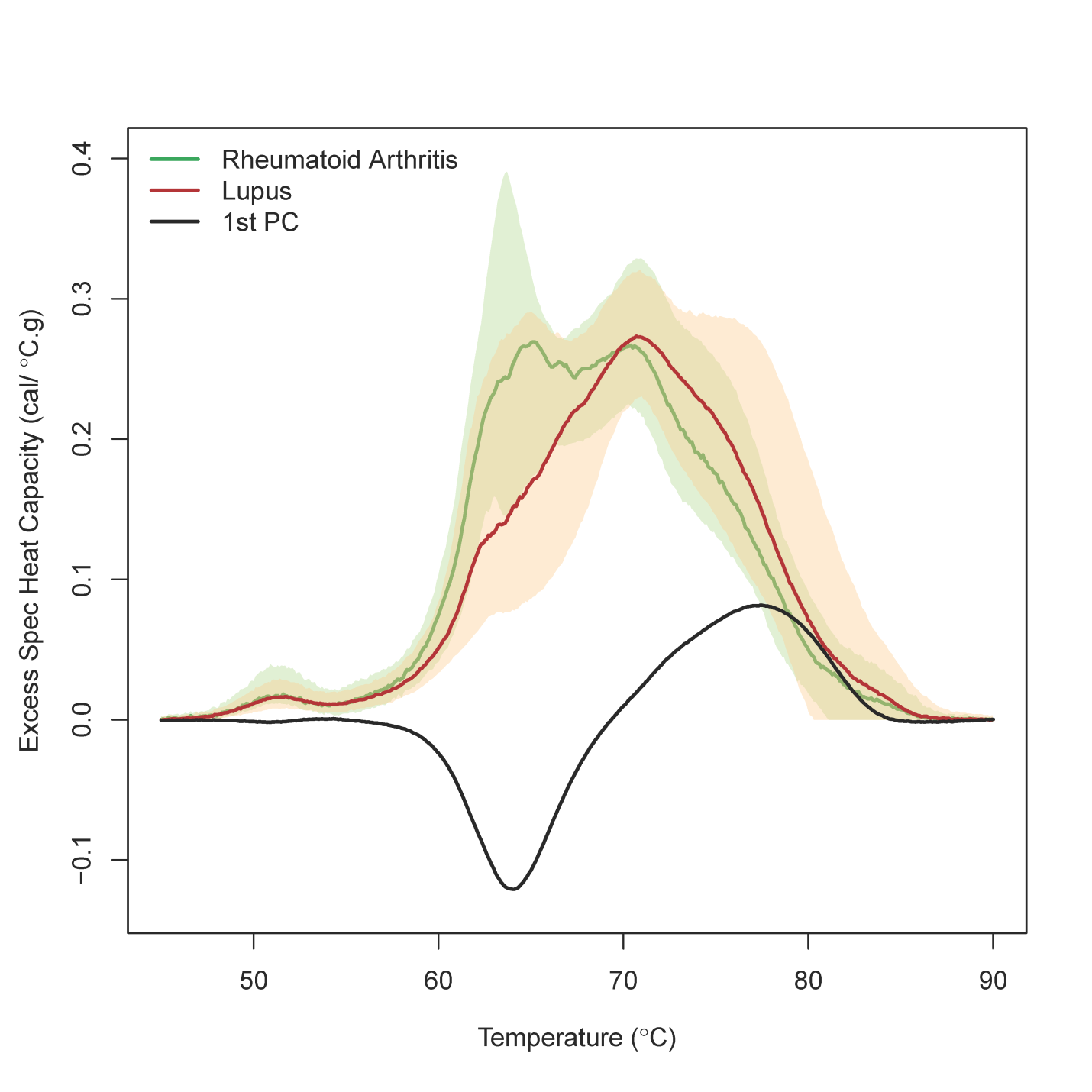

Supplement: S2 Fig — Bands represent the 5th and 95th percentiles among subjects at each temperature. The loadings for the first principal component among all subjects are shown as the black line. (TIF) [file pone.0186398.s006.tif]

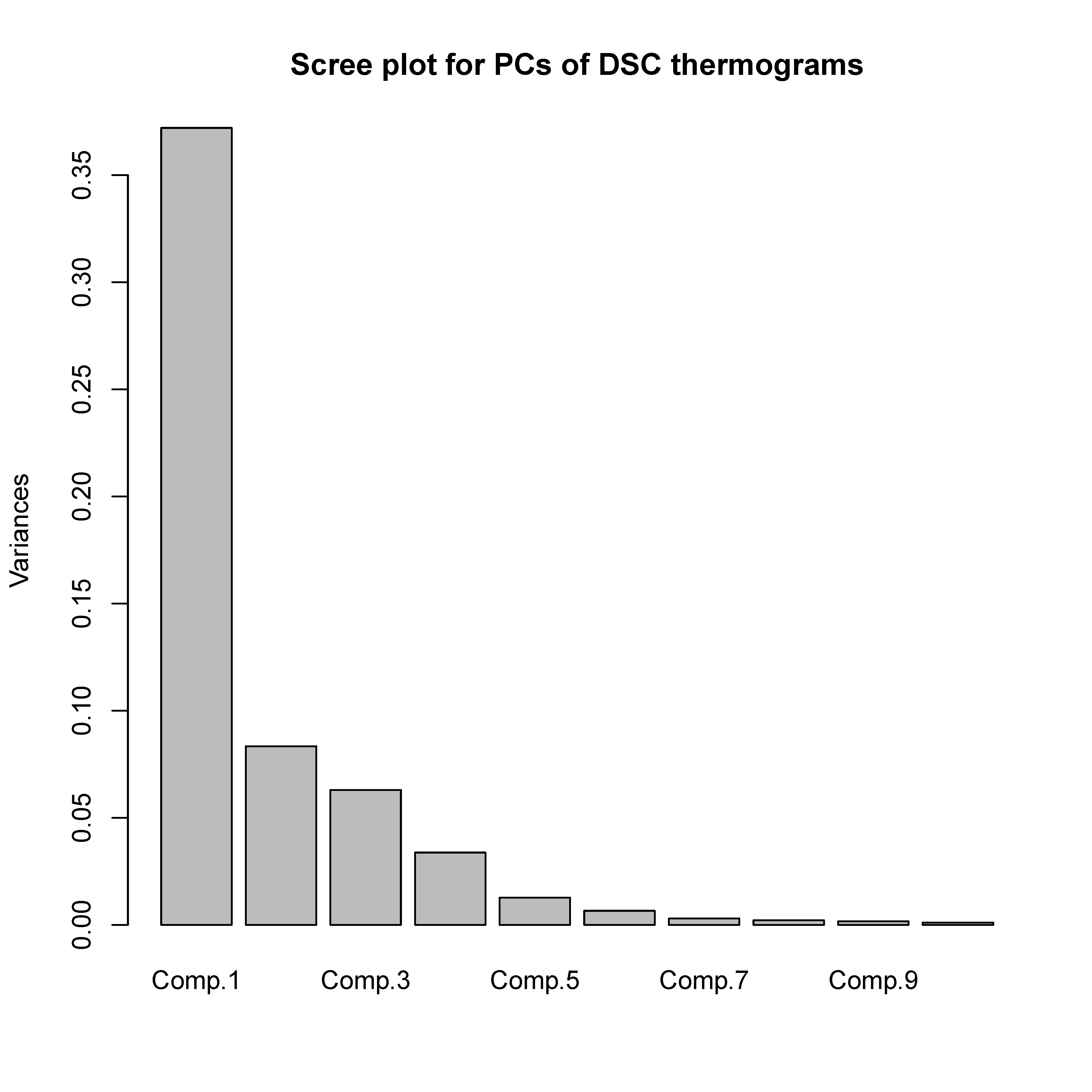

Supplement: S3 Fig — (TIF) [file pone.0186398.s007.tif]

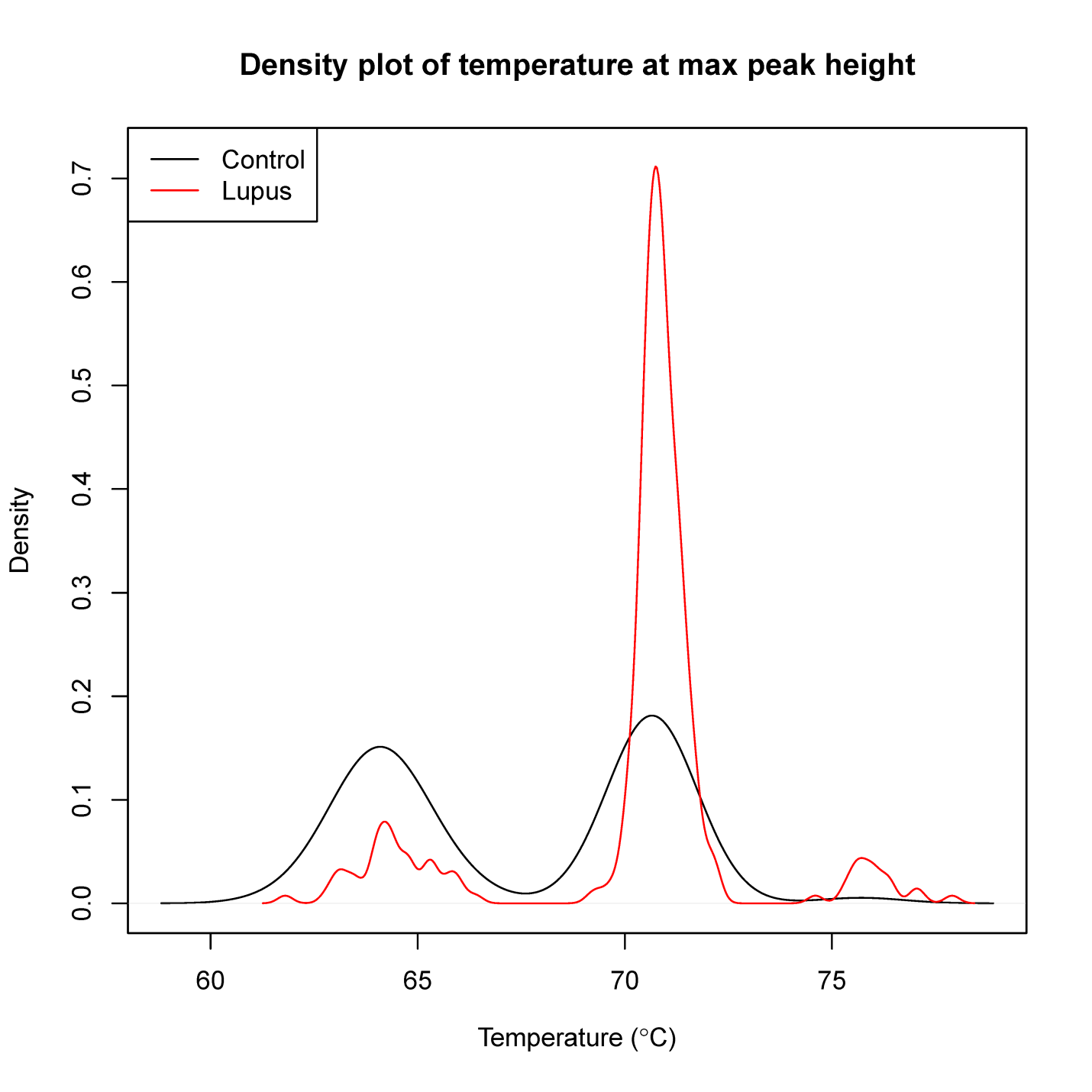

Supplement: S4 Fig — The density plots reveal roughly three prominent peaks among the subjects at 62–67°C, 69–73°C, and 75–80°C (the latter being present only among lupus patients). (TIF) [file pone.0186398.s008.tif]

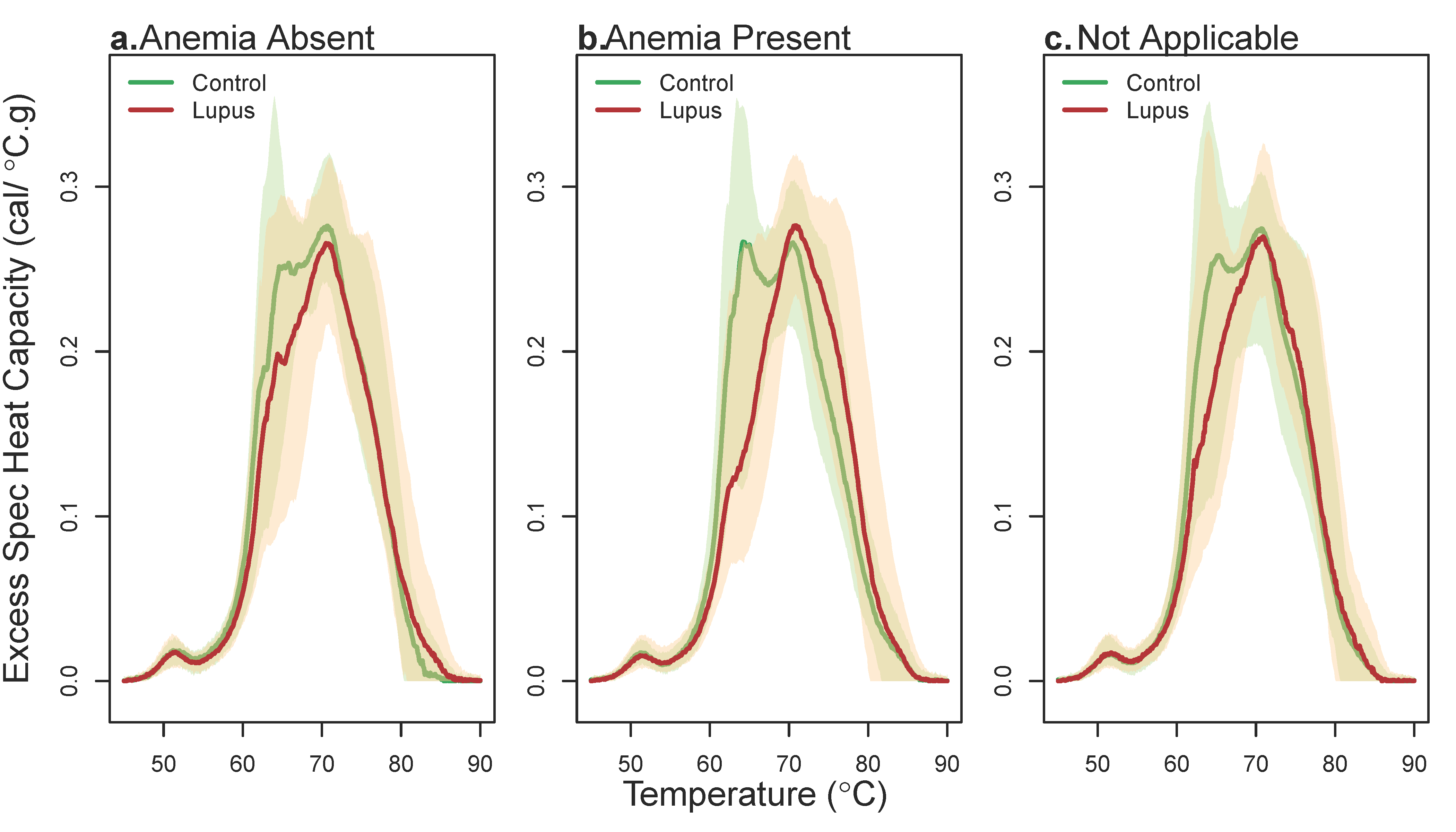

Supplement: S5 Fig — Not applicable indicates that the study question did not apply. Bands represent the 5th and 95th percentiles among subjects at each temperature. (TIF) [file pone.0186398.s009.tif]

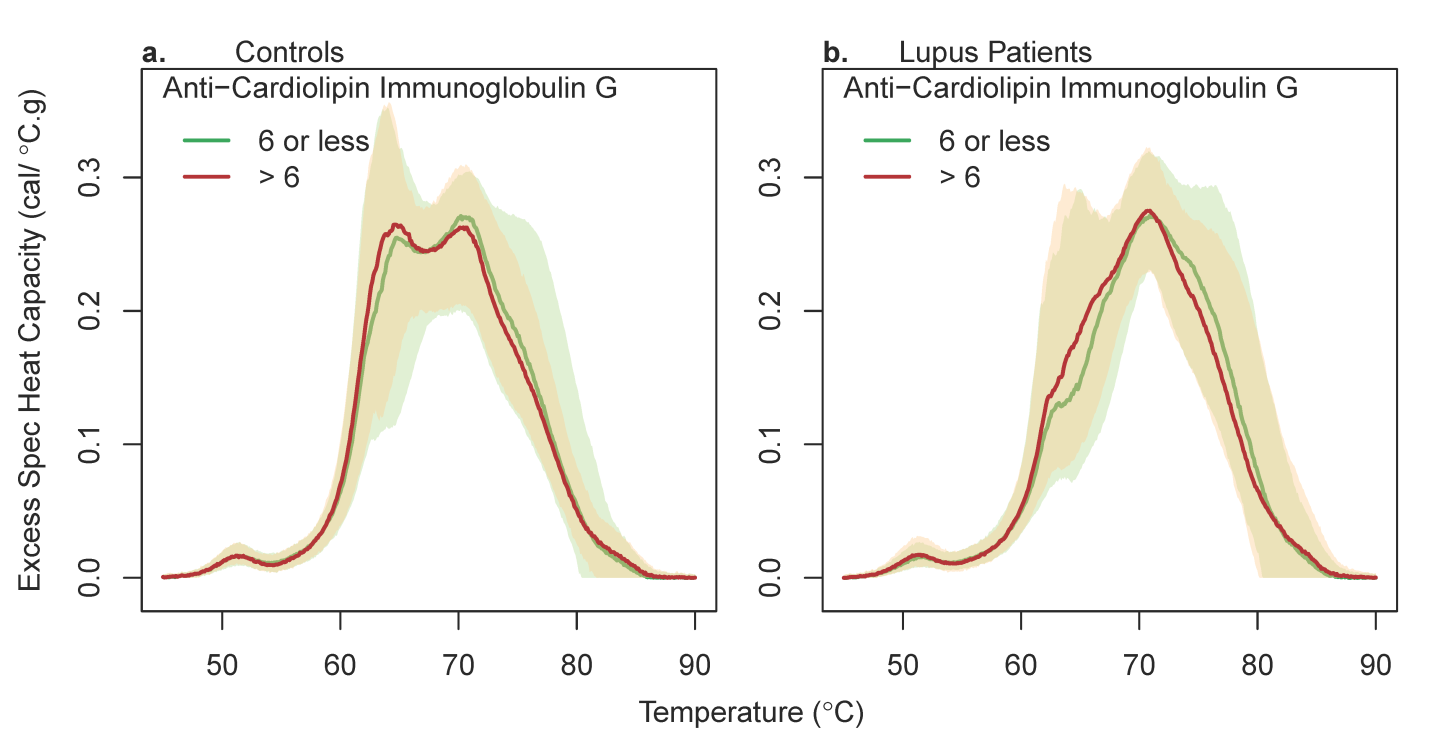

Supplement: S6 Fig — Cut-point at the median value of 6). Bands represent the 5th and 95th percentiles among subjects at each temperature. (TIF) [file pone.0186398.s010.tif]

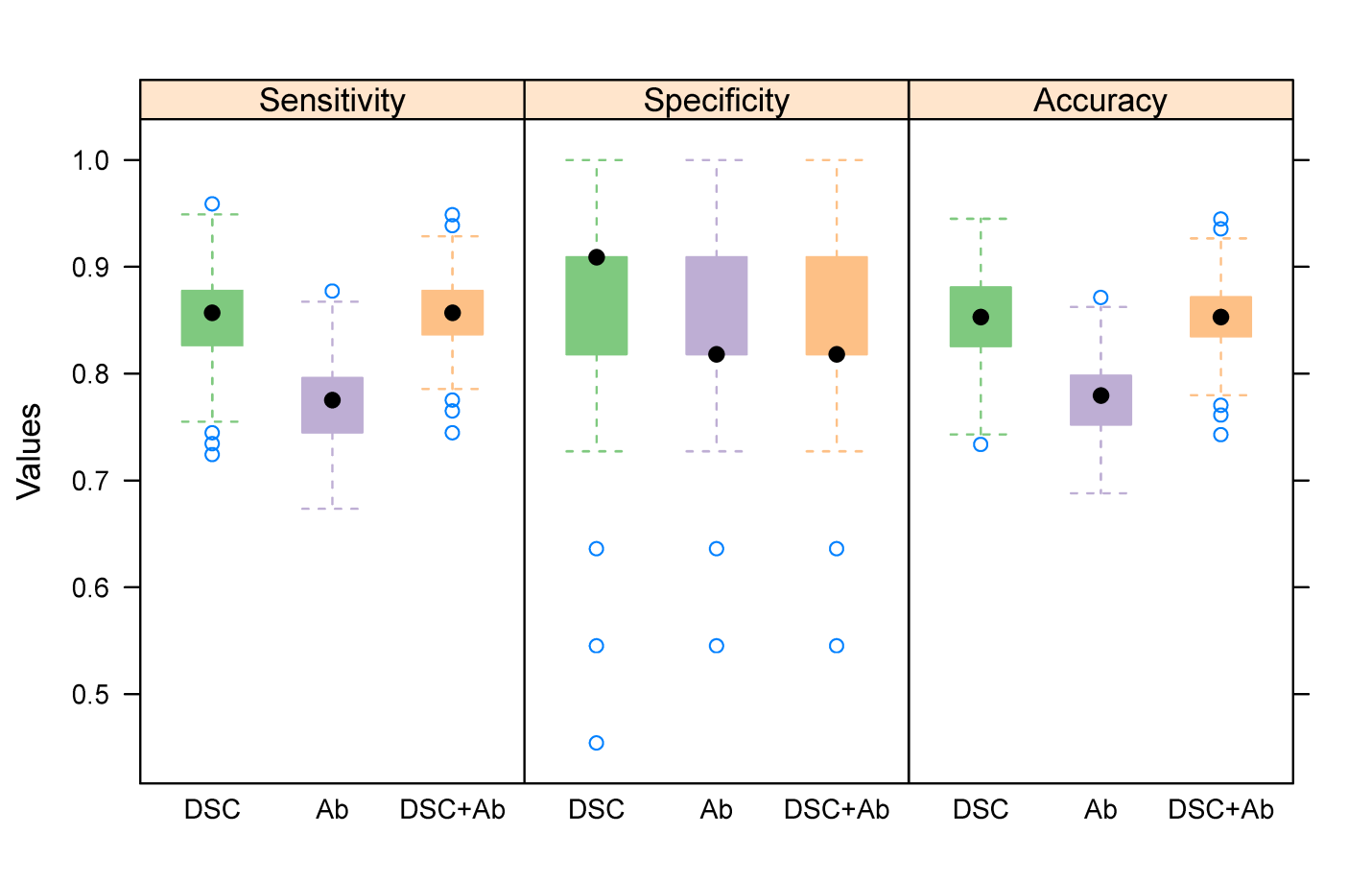

Supplement: S7 Fig — Boxplots represent values from 1000 test data sets created by splitting the data randomly into training (two thirds) and testing (one third) sets. (TIF) [file pone.0186398.s011.tif]

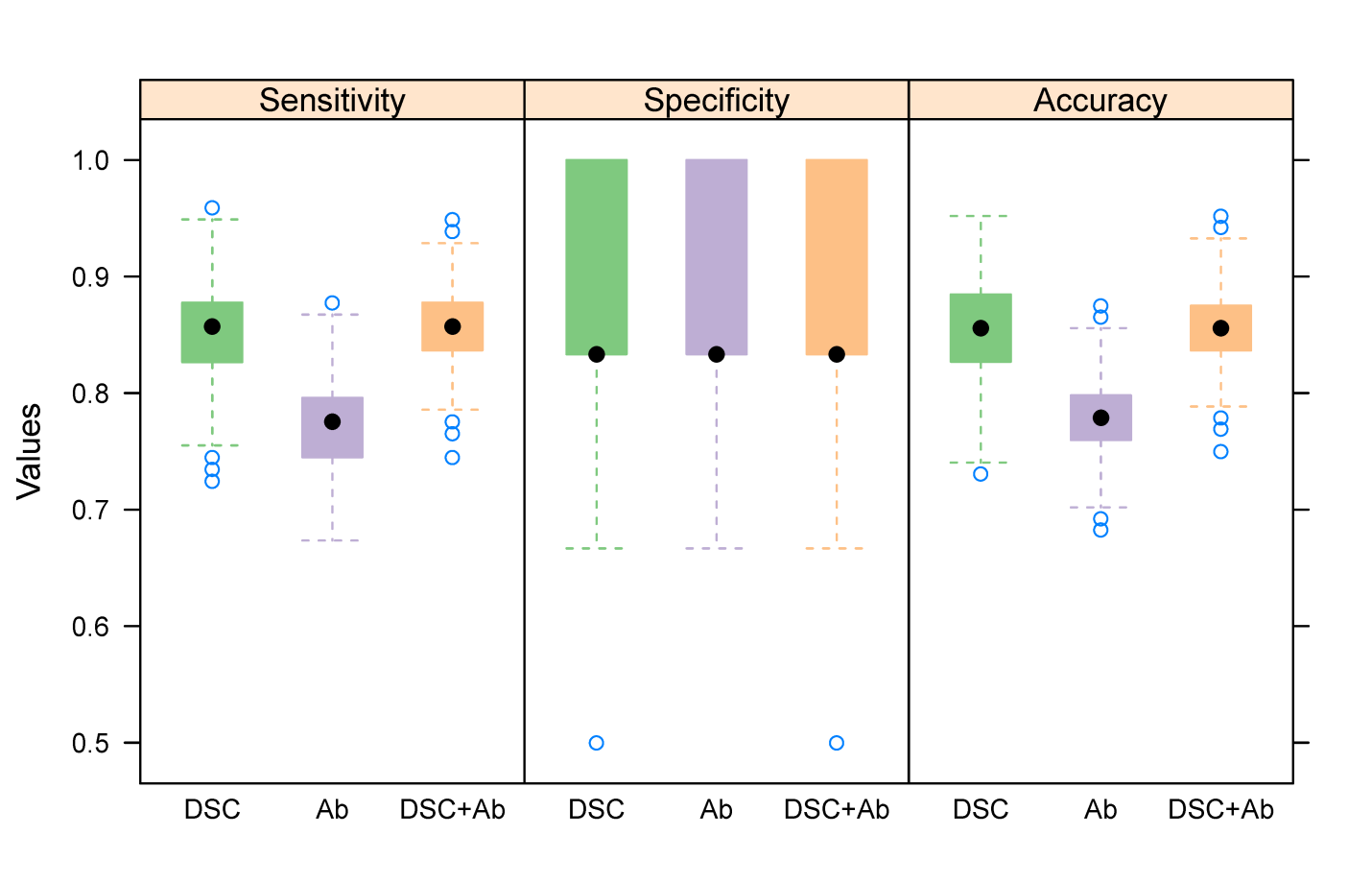

Supplement: S8 Fig — Boxplots represent values from 1000 test data sets created by splitting the data randomly into training (two thirds) and testing (one third) sets. (TIF) [file pone.0186398.s012.tif]
